# Ceria-Based Nanotherapeutics for Inflammatory Skin Disorders: From Design and Mechanisms to Advanced Delivery and Future Perspectives

**DOI:** 10.3390/ijms27188243

**Published:** 2026-09-16

**Authors:** Zishang Guo, Wenshang Liu, Fei Pan, Fangwei Guo, Dan Deng

**Affiliations:** 1 Department of Dermatology, Shanghai Children’s Medical Center, Shanghai Jiao Tong University School of Medicine, Shanghai 200127, China; eirikkguo@sjtu.edu.cn (Z.G.); sixwinsong@163.com (W.L.); 2 Department of Health Science and Technology, ETH Zürich, 8092 Zurich, Switzerland; phoenix.pan@tum.de; 3 Shanghai Key Laboratory of Advanced High-Temperature Materials and Precision Forming, School of Materials Science and Engineering, Shanghai Jiao Tong University, Shanghai 200240, China

**Keywords:** cerium oxide nanoparticles, inflammatory skin diseases, enzyme-mimetic activity, structure–activity relationship, advanced delivery strategies

## Abstract

Inflammatory skin diseases, including atopic dermatitis (AD), psoriasis, diabetic wounds, and photoaging, represent major public health challenges due to their high prevalence, chronic recurrence, and substantial disease burden. Current treatments, such as glucocorticoids, calcineurin inhibitors, and biologics, are often constrained by limited efficacy, long-term adverse effects, or high costs, highlighting the need for safer and more accessible therapeutic strategies. Cerium oxide nanoparticles (CeO_2_ NPs) have attracted increasing attention because of their reversible Ce^3+^/Ce^4+^ redox cycling and multiple enzyme-mimetic activities. This review examines the synthesis strategies and structure–activity relationships of CeO_2_ NPs, and discusses their antioxidant, anti-inflammatory, immunomodulatory, antimicrobial, and proangiogenic mechanisms in inflammatory skin microenvironments. We further evaluate their applications in AD, psoriasis, diabetic wounds, and photoaging, and summarize advanced delivery approaches involving structural functionalization, biomimetic surface modification, and formulation hybridization. Key translational challenges, including long-term safety, degradability, manufacturing consistency, and disease-specific applicability, are also discussed. Finally, we propose a “5D” framework for next-generation CeO_2_-based nanomedicines, integrating disease-specific design, degradability, data-driven optimization, device integration, and digital health interfacing.

## 1. Introduction

Inflammatory skin disorders, such as atopic dermatitis (AD), psoriasis, diabetic wounds, and photoaging, have emerged as major public health challenges worldwide. Epidemiological evidence highlights the substantial health and economic burdens associated with these conditions. According to the Global Burden of Disease Study, AD alone accounted for 5.62 million disability-adjusted life years and 16 million new cases globally in 2021, ranking 15th among all nonfatal diseases in terms of disease burden [1]. Similarly, psoriasis, another prevalent immune-mediated dermatosis, has shown an increasing global impact. In China, for example, the age-standardized prevalence of psoriasis increased significantly from 362.04 per 100,000 population in 1990 to 474.02 per 100,000 population in 2021 [2]. These diseases not only substantially impair patients’ quality of life but also impose considerable socioeconomic burdens. For example, data from European countries indicate that the average annual total cost per patient with AD can reach €4331, with productivity losses accounting for more than 50% of this amount [1]. Current clinical treatment options include topical corticosteroids, calcineurin inhibitors, and biologic agents; however, several critical limitations remain. Although immunosuppressive therapies can effectively alleviate disease activity, their long-term use may be associated with cumulative toxicities, including nephrotoxicity, hepatotoxicity, and myelosuppression [3]. Biologic agents provide targeted therapeutic effects but remain constrained by high costs and limited accessibility, particularly for patients with mild-to-moderate disease [4]. Furthermore, most existing therapies are unable to achieve a definitive cure, often necessitating long-term management of recurrent symptoms. These challenges highlight the urgent clinical need for novel strategies that are both effective and affordable, a gap that nanomedicine is well-suited to address.

Cerium oxide nanoparticles (CeO_2_ NPs), a class of rare-earth metal oxide nanozymes, exhibit remarkable potential for the treatment of inflammatory skin diseases because of their unique reversible Ce^3+^/Ce^4+^ redox switching capability [5,6]. First, CeO_2_ NPs are capable of mimicking both superoxide dismutase (SOD) and catalase (CAT) activity through the reversible redox cycling of surface Ce^3+^ and Ce^4+^ species, thereby enabling the elimination of the cascade of superoxide anions, hydrogen peroxide, and hydroxyl radicals [7]. This broad-spectrum antioxidant capacity substantially surpasses that of single-function natural enzymes or small-molecule antioxidants. Second, CeO_2_ NPs exhibit pronounced pH- and redox-responsive behavior, maintaining robust catalytic activity even within the inflamed skin microenvironment, which is characterized by a weakly acidic environment and reactive oxygen species (ROS) [6]. This characteristic addresses a critical limitation of most natural enzymes, which typically function optimally under neutral conditions but readily lose activity in pathological microenvironments. Third, the surface of CeO_2_ NPs can be readily functionalized, enabling stable conjugation with intrinsic skin components such as stratum corneum lipids, hyaluronic acid, and antimicrobial peptides [6]. Such versatility provides a robust platform for topical delivery systems, facilitating targeted deposition at lesion sites and prolonged retention. Finally, when combined with biomaterials such as hydrogels, microneedles, or microspheres, CeO_2_ NPs offer synergistic advantages for localized and sustained delivery, by providing a moist wound environment, establishing microchannels, or enabling controlled release, thereby achieving precise drug administration [8,9,10]. CeO_2_ NPs integrate a triad of functionalities, including multienzyme activity, microenvironmental adaptability, and surface programmability/composability, making them well-suited to the complex pathophysiology of inflammatory skin diseases, which are characterized by oxidative stress, immune dysregulation, and barrier disruption. As an ideal nanoplatform that combines broad-spectrum antioxidant activity, precise immunomodulation, and synergistic local delivery, CeO_2_ NPs hold substantial promise for treating skin inflammatory disorders.

This review aims to synthesize recent research on CeO_2_ NPs in the context of inflammatory skin diseases, offering systematic guidance for their safe, rational, and efficient development. The article is organized around four core themes. First, it systematically examines synthetic methods and surface engineering strategies for CeO_2_ NPs, elucidating how parameters such as size, morphology, crystal facets, doping, and surface modification influence their antioxidant activity and biological functions. Second, it explores the multitarget biological mechanisms of CeO_2_ NPs within the cutaneous microenvironment, including the antioxidant protection of keratinocytes, immunomodulation of macrophages and T-cell subsets, antimicrobial activity against bacteria and biofilms, and promotion of angiogenesis. Third, it summarizes advanced delivery strategies based on structural functionalization, surface biomimetic modification, and formulation hybridization, including microneedles, hydrogels, and microspheres, and discusses how these approaches enable precise targeting, controlled release, and synergistic therapy at lesion sites. Finally, key bottlenecks in clinical translation are examined, and future directions for “5D” development trends (disease-specific, degradable, data-driven, device-integrated, and digital health) for next-generation CeO_2_-based nanomedicines are proposed. By establishing an interdisciplinary dialog framework encompassing materials, mechanisms, delivery, and translation, this review aims to provide a shared technical language for dermatologists, nanomaterial researchers, and pharmaceutical developers, serving as a reference for the rational design and clinical translation of ceria-based nanomedicines.

## 2. Design Strategies for CeO_2_ NPs

### 2.1. Synthesis Methods of Cerium Oxide Nanoparticles

The synthesis method of CeO_2_ NPs critically affects their final properties. Given that the Ce^3+^/Ce^4+^ ratio and surface oxygen vacancy concentration directly govern the catalytic activity and biological functions of these nanozymes, substantial efforts have recently been directed toward modulating synthetic approaches [11]. Table 1 provides a systematic summary and comparison of the current mainstream synthesis techniques.

The antioxidant properties of CeO_2_ NPs are inherently dependent on the presence and distribution of oxygen vacancies on their surface. These oxygen vacancies enable reversible redox cycling between Ce^3+^ and Ce^4+^, thereby determining the catalytic activity of these nanozymes [18,19]. Consequently, precise regulation of the Ce^3+^/Ce^4+^ ratio and surface oxygen vacancy concentration is key to enhancing the performance of CeO_2_ NPs, with different synthesis methods resulting in significant differences [20,21]. For example, when CeO_2_ NPs are prepared via wet chemical synthesis or hydrothermal methods, the introduction of various ligands can effectively modulate the surface oxygen vacancy content and the Ce^3+^/Ce^4+^ ratio, thereby altering their enzyme-mimetic activity [22]. Additionally, doping techniques are widely used to adjust the oxygen vacancy concentration and the Ce^3+^/Ce^4+^ ratio, thereby further improving the material’s electrochemical properties and catalytic activity [23,24]. Engineering design based on oxygen vacancies not only enhances the SOD- and CAT-mimetic activities of CeO_2_ NPs, thereby effectively modulating the microenvironment in oxidative stress-related diseases, but also promotes cellular uptake and ROS scavenging, ultimately increasing their potential for biomedical applications [25]. Furthermore, the regulation of oxygen vacancies directly affects the stability and biocompatibility of CeO_2_ NPs in biological environments, laying the foundation for their safe and effective application [21,26].

Therefore, differences in synthesis methods for cerium oxide nanoparticles, particularly in their ability to modulate the Ce^3+^/Ce^4+^ ratio and surface oxygen vacancy concentration, directly determine their catalytic activity as nanozymes. This characteristic not only influences the application of CeO_2_ NPs in antioxidant and anti-inflammatory therapies but also opens new avenues for their further development in the biomedical field.

### 2.2. Structure–Activity Relationships of CeO_2_ NPs

**Figure 1:**
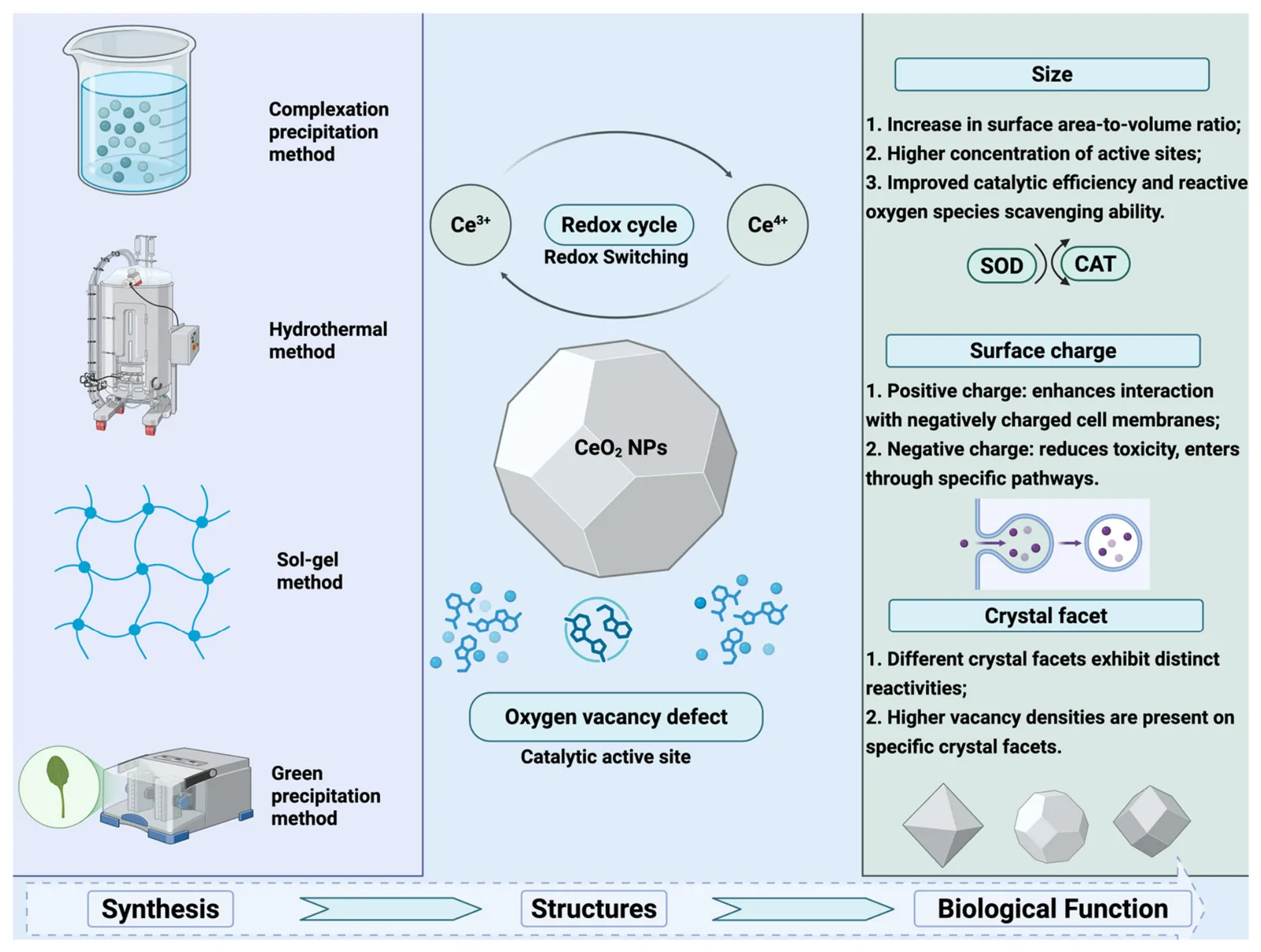
Preparation method of cerium oxide nanoparticles (CeO_2_ NPs) and the factors affecting their activity. The circular arrows between Ce^3+^ and Ce^4+^ indicate the reversible redox cycling of cerium ions, while the arrows between SOD and CAT indicate the coordinated SOD- and CAT-like antioxidant activities of CeO_2_ NPs. Created in BioRender. Liu, W. (2026) https://BioRender.com/nuhgrdw (accessed on 13 September 2026).

The structure–activity relationships of CeO_2_ NPs are a key focus in nanotechnology and biomedical research. Factors such as particle size, surface charge, crystal phase, and surface modification significantly influence the activity of CeO_2_ NPs. The preparation method of the CeO_2_ NPs and the factors affecting their activity are shown in Figure 1.

First, particle size plays a critical role in governing catalytic activity. Smaller particles generally exhibit higher catalytic activity because of their larger specific surface area and higher surface Ce^3+^ concentration [27], which collectively enhance their functional performance. Moreover, the size of CeO_2_ NPs also affects cellular uptake and toxicity; smaller particles are more readily internalized by cells and may exhibit enhanced cytotoxicity in certain contexts [28].

Second, the surface charge of cerium oxide nanoparticles also significantly influences their biological activity. Surface charge differences govern nanoparticle cellular internalization and subcellular localization, resulting in differential toxicity: positively or neutrally charged particles, although readily internalized by multiple cell types, display marked toxicity only when they localize to cancer cell lysosomes, whereas negatively charged particles are selectively taken up by cancer cells and reside in lysosomes, exerting little to no toxicity toward normal cells [29]. Moreover, surface modification can modulate biological activity by altering particle charge and surface properties. For instance, CeO_2_ NPs modified with a nonionic, sugar-based surfactant, dodecyl maltoside, exhibit increased antiamyloid activity while still retaining some antioxidant potential [30].

Finally, the crystal phase and surface modification of cerium oxide nanoparticles are also critical determinants of their activity. Studies indicate that the crystal phase of CeO_2_ NPs can influence their catalytic activity and biocompatibility by modulating the distribution of oxygen vacancies [31]. In addition, surface modification enables regulation of particle bioactivity by introducing different functional groups. For example, CeO_2_ NPs modified with catecholate-type ligands exhibit enhanced biomedical potential due to the formation of interfacial charge-transfer complexes [32].

The structure–activity relationships of CeO_2_ NPs elucidate the core principles underlying their rational design as nanozymes: structural parameters, including size, surface charge, crystal phase, and surface modification, collectively govern their catalytic activity, biocompatibility, and functional specificity. Particle size directly modulates SOD/CAT-mimetic activities and cellular uptake behavior by influencing the specific surface area and surface Ce^3+^ concentration. Surface charge dictates cellular localization and internalization pathways, thereby shaping downstream biological outcomes. The crystal phase regulates catalytic efficiency and biocompatibility by altering the distribution of oxygen vacancies. Moreover, surface modification not only alters surface charge properties but also confers additional biological functions by introducing functional ligands. Consequently, the activity of CeO_2_ NPs is not dictated by any single structural factor but emerges from the synergistic interplay of multiple parameters. A thorough understanding and precise regulation of this structure–activity relationship represent critical prerequisites for advancing their clinical translation and achieving optimized targeted antioxidant and immunomodulatory functions.

## 3. Biological Properties of CeO_2_ NPs

**Figure 2:**
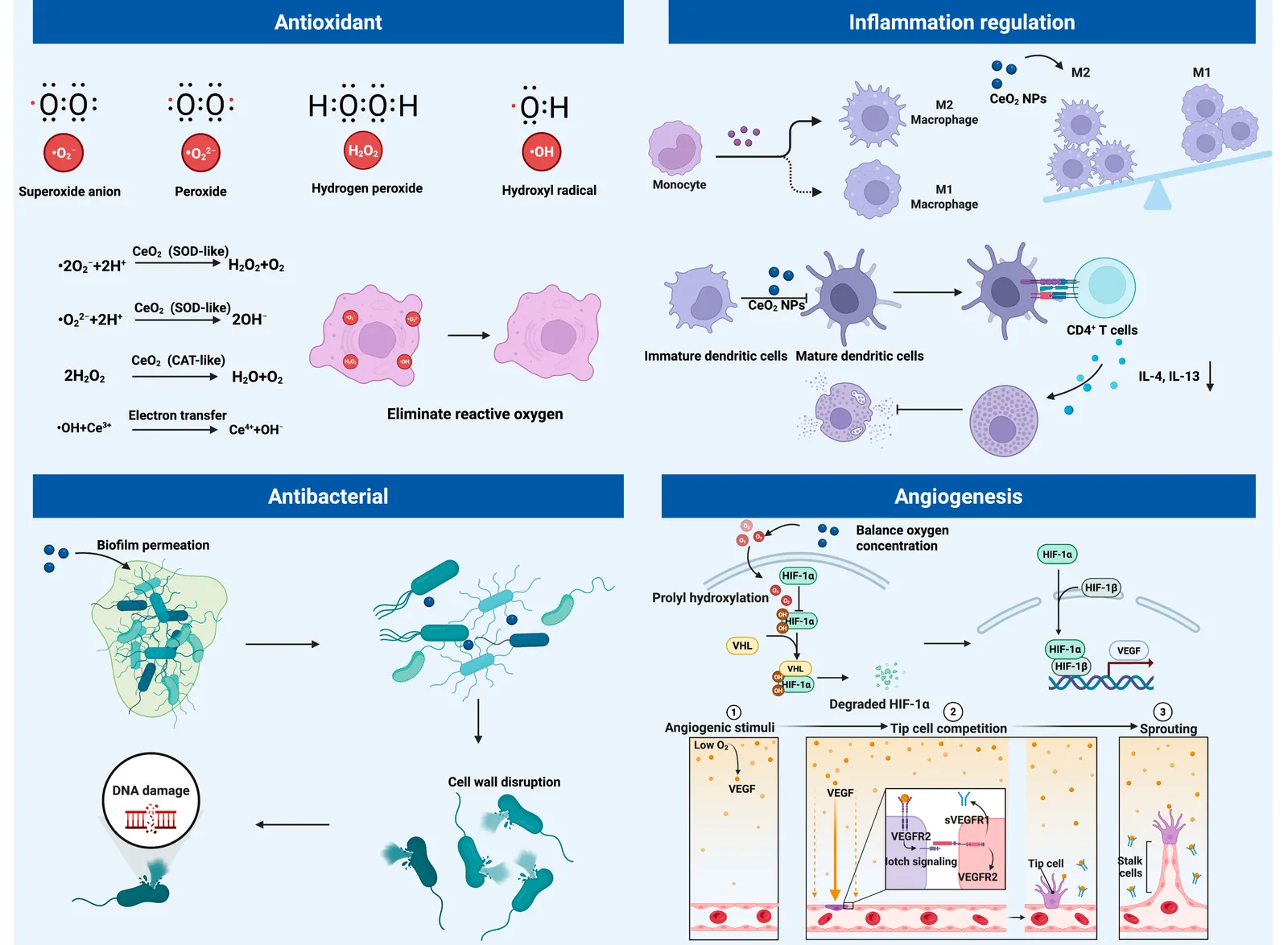
Schematic diagram of the biological functional mechanisms of CeO_2_ NPs. Created in BioRender. Liu, W. (2026) https://BioRender.com/0u3wesv (accessed on 13 September 2026).

The biological functions of CeO_2_ NPs arise from the multienzyme-mimetic activities conferred by their unique structure. This structure–activity relationship underpins their application in complex skin microenvironments. In the treatment of inflammatory skin diseases, the core biological properties of CeO_2_ NPs are manifested primarily in multiple ways, including the scavenging of ROS, regulation of inflammatory and immune cell functions, inhibition of pathogenic microorganisms, and promotion of tissue repair, thereby enabling multifaceted intervention in the pathological processes of the disease (Figure 2).

### 3.1. Antioxidant Activity

The core antioxidant advantage of CeO_2_ NPs stems from their unique electronic structure and quantum-size effects. This characteristic offers notable benefits, particularly in skin care: upon exposure to ultraviolet (UV) irradiation, keratinocytes generate substantial amounts of ROS, leading to oxidative stress [33]. The abundant oxygen vacancy defects on the surface of CeO_2_ NPs enable rapid, reversible, and dynamic switching between the Ce^3+^ and Ce^4+^ oxidation states, thereby effectively scavenging excess ROS and preserving cell viability [34].

Furthermore, CeO_2_ NPs can be conceptualized as “electron reservoirs” that participate in intracellular electron transfer via surface redox cycling, thereby modulating the redox balance of skin cells [35]. More importantly, their antioxidant activity is characterized by catalytic regenerability: conventional sacrificial small-molecule antioxidants (e.g., vitamin C and vitamin E) are consumed upon the scavenging of ROS and require continuous replenishment. In contrast, via the dynamic equilibrium of surface Ce^3+^/Ce^4+^ ratios, CeO_2_ NPs can self-regulate during ROS scavenging, thereby regenerating their catalytic activity and conferring sustained protection to the skin [36].

Various parameters, including particle size, morphology, and surface modification, precisely modulate the antioxidant efficacy of CeO_2_ NPs. Owing to their relatively large specific surface area, ultrasmall nanoparticles maximize surface Ce^3+^/Ce^4+^ exchange and oxygen vacancy formation, thereby increasing their redox activity [37]. Through surface engineering, for example, coating with citric acid or functionalization with dopamine to introduce ortho-quinone structures, their aqueous dispersibility, biocompatibility, and intracellular antioxidant efficiency can be further improved [38]. Furthermore, integrating CeO_2_ NPs with polymers such as poly(maleic anhydride-alt-1-octadecene)-Jeffamine not only prevents aggregation but also preserves their autocatalytic properties, thereby achieving stability across a broad pH range and conferring selective cytotoxicity [39].

Based on the aforementioned advantages, CeO_2_ NPs hold significant promise for skin care applications. They not only effectively alleviate UV-induced oxidative stress in skin cells [40] but also promote skin barrier repair and delay skin aging. Furthermore, they can serve as carriers for active ingredients, enabling targeted delivery and synergistic effects [41].

### 3.2. Immunomodulatory Effects

In addition to their antioxidant function, CeO_2_ NPs have been reported to modulate the activity and phenotypic states of multiple immune cell populations. By scavenging excess ROS in the microenvironment, these nanoparticles can interfere with the activation of proinflammatory signaling pathways such as nuclear factor kappa B (NF-κB), thereby attenuating proinflammatory M_1_ macrophage activation and reducing the transcription and release of inflammatory mediators, including tumor necrosis factor-alpha (TNF-α), interleukin-6 (IL-6), inducible nitric oxide synthase, and nitric oxide, while creating conditions that may favor macrophage polarization toward an anti-inflammatory M_2_ phenotype [42]. Consistent with this effect, CeO_2_ NP treatment has been reported to upregulate the expression of M_2_ markers, including arginase-1, IL-10, IL-4, and transforming growth factor-beta (TGF-β) [42]. In AD models, porous cerium oxide nanorods significantly reduced ROS levels in TNF-α-stimulated keratinocytes and lipopolysaccharide-stimulated M_1_ macrophages, and modulated the expression of key inflammatory cytokines [5]. Collectively, these findings suggest that CeO_2_-mediated modulation of the oxidative and inflammatory microenvironment can influence macrophage activation and phenotypic remodeling.

Furthermore, CeO_2_ NPs have been reported to modulate adaptive and allergic immune responses. In AD models, CeO_2_ NP treatment attenuated T helper 2 (Th2)-dominant inflammation, as evidenced by reduced Th2-cell activation, decreased levels of IL-4, IL-13, and serum immunoglobulin E levels, and reduced mast-cell infiltration and scratching behavior [5]. CeO_2_ NPs have also been shown to suppress antigen-specific CD8^+^ T-cell activation, primarily through modulation of ROS-mediated T-cell activation signals rather than through direct effects on antigen-presenting cells [43]. Transcriptomic analyses further revealed that CeO_2_ NPs systematically downregulated genes associated with cell activation and innate immunity, including *Fos*, *Trpm2*, *Cybb*, and *Nlrc4* [44]. In addition, by modulating ROS signals involved in mast-cell activation within the local microenvironment, CeO_2_ NPs may reduce mast-cell degranulation and histamine release, thereby contributing to the alleviation of pruritus in allergic skin inflammation [5].

Nevertheless, these observations should not be interpreted as evidence that ROS scavenging inherently produces selective immunosuppression. At physiological levels, ROS function as important second messengers involved in immune-cell activation, signal transduction, and host defense, whereas excessive or sustained ROS accumulation can amplify inflammatory signaling and cause oxidative damage [45]. The biological consequences of ROS modulation are therefore dependent on the concentration, source, duration, and subcellular localization of ROS, as well as the responding cell type and surrounding microenvironment [45,46]. Accordingly, the immunomodulatory effects of CeO_2_ NPs are likely to be dose- and context-dependent rather than intrinsically M_2_-polarizing or selectively suppressive toward particular T-cell subsets. Supporting this context dependency, CeO_2_ NPs with different morphologies have been reported to differentially regulate ROS/RNS levels and macrophage phenotypes, with cube-shaped particles promoting an M_1_-associated phenotype in an experimental respiratory syncytial virus infection model [47]. These findings suggest that nanoparticle physicochemical characteristics and disease-specific inflammatory cues can substantially influence the resulting immune response. Thus, the immunomodulatory activity of CeO_2_ NPs may be more appropriately understood as modulation of pathological redox imbalance rather than indiscriminate elimination of ROS.

Moreover, the observed changes in macrophage polarization, Th2-associated responses, and CD8^+^ T-cell activation do not necessarily establish a broadly applicable mechanism of direct and selective immune-cell targeting by CeO_2_ NPs. Although existing studies provide evidence that CeO_2_ NPs can modulate specific immune responses, some of these effects may arise indirectly from attenuation of oxidative stress and broader remodeling of the inflammatory microenvironment. The relative contributions of direct immune-cell regulation and indirect microenvironment-mediated effects therefore remain insufficiently defined. Future studies using purified immune-cell populations, keratinocyte–immune cell co-culture systems, dose–response designs, and cell-specific mechanistic analyses will be important for establishing whether and under what conditions CeO_2_ NPs directly regulate individual immune-cell subsets.

In summary, current evidence indicates that CeO_2_ NPs can modulate macrophage, T-cell, and mast-cell responses in association with changes in the local redox and inflammatory microenvironment. CeO_2_-mediated attenuation of excessive ROS has been associated with reduced M_1_-like inflammatory activity and, in some experimental settings, a shift toward anti-inflammatory or reparative macrophage phenotypes. CeO_2_ NPs have also been reported to attenuate Th2-associated allergic responses, antigen-specific CD8^+^ T-cell activation, and mast-cell degranulation. However, these findings should be interpreted as context-dependent immunomodulatory effects rather than universal or intrinsically cell-selective actions. Future studies should therefore define dose–response relationships and disease-specific redox contexts and distinguish direct effects on individual immune-cell subsets from indirect effects arising from redox normalization and microenvironmental remodeling.

### 3.3. Antimicrobial Activity

Owing to the unique electronic structure and interfacial properties of CeO_2_ NPs, their antimicrobial mechanisms can be systematically delineated into four interrelated aspects: intrinsic enzyme-mimetic catalysis, physical membrane interactions, structural penetration and biofilm degradation, and multidimensional modulation of the infectious microenvironment. Rather than operating in isolation, these mechanisms act synergistically across multiple scales, collectively constituting a multilevel, multitarget antimicrobial system.

First, at the molecular level, the antimicrobial activity of CeO_2_ NPs is fundamentally associated with their enzyme-mimetic catalytic properties, which enable regulation of ROS under acidic or oxidative stress conditions. As the particle size decreases or the proportion of Ce^3+^ increases, catalytic activity can increase, allowing both the generation of ROS that induce oxidative damage to bacterial lipids, proteins, and deoxyribonucleic acid (DNA) and the modulation of local oxygen tension via enzyme-like activity, thereby indirectly inhibiting bacterial metabolism and proliferation [48,49]. Together, these features establish a molecular-level basis for the antimicrobial activity of CeO_2_ NPs.

Second, at the cellular level, CeO_2_ NPs can disrupt bacterial structural integrity through physical interfacial interactions. Positively charged nanoparticles interact with negatively charged bacterial surfaces through electrostatic adsorption, which can perturb membrane potential and compromise membrane integrity. Strategies such as copper modification can further enhance physical insertion into and disruption of the Gram-positive bacterial cell wall [50]. Moreover, CeO_2_ NPs with porous foam architectures exhibit improved separation efficiency of photogenerated electron–hole pairs under light irradiation, facilitating ROS generation and enhancing bactericidal efficacy [51]. Collectively, these mechanisms underscore the critical role of surface characteristics and structural engineering in determining antimicrobial performance.

Third, in the context of biofilm-associated refractory infections, CeO_2_ NPs exhibit multifaceted intervention capabilities, including biofilm penetration and extracellular matrix degradation. Cationically modified composites can traverse the biofilm barrier, inhibiting planktonic bacterial growth while penetrating deeper into the biofilm, where they disrupt the matrix by degrading extracellular polysaccharides and DNA [52]. Their DNase-like activity can further hydrolyze extracellular DNA, thereby compromising biofilm structural integrity. This integrated mode of action, combining physical penetration, chemical degradation, and enzymatic hydrolysis, highlights the potential of CeO_2_-based systems for managing biofilm-associated infections.

Finally, at the tissue scale, CeO_2_ NPs can modulate the pathological microenvironment of infected skin by integrating antimicrobial, anti-inflammatory, and reparative effects. Chronic infected wounds, including diabetic foot ulcers, are frequently characterized by persistent inflammation, oxidative stress imbalance, and impaired angiogenesis. Pluronic F127-functionalized CeO_2_ NPs not only reduced bacterial burden at the wound site but also significantly downregulated proinflammatory factors such as TNF-α and IL-6 while concurrently upregulating vascular endothelial growth factor (VEGF) expression, thereby promoting angiogenesis and facilitating tissue remodeling [49]. Such findings suggest that CeO_2_-based antimicrobial systems may provide benefits beyond direct bacterial inhibition by simultaneously modulating multiple components of the wound microenvironment.

Despite these promising antimicrobial effects, broad-spectrum antimicrobial activity should not necessarily be regarded as uniformly beneficial in cutaneous applications. Healthy skin harbors diverse commensal microbial communities that contribute to colonization resistance, epidermal barrier homeostasis, and regulation of local immune responses [53]. Consequently, antimicrobial interventions may affect not only pathogenic microorganisms but also beneficial resident bacteria. Indeed, topical antimicrobial treatments have been shown to alter resident skin bacterial communities and reduce commensal *Staphylococcus* populations that participate in resistance to *Staphylococcus aureus* colonization [54]. Moreover, longitudinal analysis of *S. aureus* decolonization using topical antimicrobials has demonstrated sustained disruption of skin bacterial communities following treatment [55]. These observations indicate that antimicrobial efficacy in cutaneous applications should be evaluated not only in terms of pathogen reduction and host-cell compatibility but also in terms of potential effects on the composition, diversity, and functional integrity of the resident skin microbiome.

This consideration is particularly important in AD, in which microbial dysbiosis is frequently characterized by increased *S. aureus* colonization and reduced bacterial diversity, especially during disease flares [53]. Importantly, not all resident *Staphylococcus* species or strains are detrimental. Specific strains of coagulase-negative *Staphylococcus*, including *S. epidermidis* and *S. hominis*, can produce antimicrobial molecules that selectively inhibit *S. aureus*, whereas such protective antimicrobial-producing commensal strains are deficient in patients with AD [56]. The potential therapeutic importance of preserving or restoring beneficial commensals is further supported by a phase 1 randomized clinical trial in which topical administration of the human skin commensal *S. hominis* A9 reduced *S. aureus* abundance in patients with AD [57]. Thus, indiscriminate suppression of resident bacteria could remove protective microbial functions and potentially further destabilize an already dysbiotic cutaneous ecosystem.

At present, whether CeO_2_ NPs preferentially suppress pathogenic bacteria while preserving beneficial skin commensals has not been sufficiently established. Therefore, the therapeutic goal of CeO_2_-based antimicrobial systems in inflammatory skin diseases should not simply be maximal or broad-spectrum microbial eradication, but rather effective control of pathogenic overgrowth while minimizing disruption of beneficial commensal communities. Future studies should directly compare CeO_2_ activity against disease-relevant pathogens and representative commensal species under identical experimental conditions and incorporate microbiome-level endpoints, including community diversity, taxonomic composition, functional changes, and recovery after repeated exposure. Such studies will be essential to determine whether CeO_2_-based antimicrobial strategies can achieve pathogen control without compromising the ecological functions of the resident skin microbiota.

In summary, the antimicrobial activity of CeO_2_ NPs involves interconnected mechanisms spanning redox catalysis, physical membrane interactions, biofilm disruption, and modulation of the infectious microenvironment. These properties support their potential for controlling pathogenic bacterial burden, particularly in infected wounds. However, in inflammatory skin diseases, antimicrobial potency alone is not an adequate measure of therapeutic value. Future CeO_2_-based strategies should balance effective suppression of pathogenic microorganisms with preservation of beneficial commensal communities and host-tissue compatibility, thereby avoiding unnecessary disruption of the cutaneous microbial ecosystem.

### 3.4. Promotion of Angiogenesis

Owing to their distinctive physicochemical properties, CeO_2_ NPs hold considerable promise for promoting angiogenesis and nutrient supply in the treatment of inflammatory skin diseases. The underlying mechanisms primarily involve three key aspects: scavenging excessive ROS to preserve endothelial cell function, stabilizing hypoxia-inducible factor-1α (HIF-1α) to upregulate VEGF expression, and modulating macrophage polarization toward the M_2_ phenotype to facilitate the secretion of tissue repair-related factors.

First, CeO_2_ NPs efficiently scavenge excess ROS in the wound microenvironment, thereby alleviating oxidative stress-induced damage to endothelial cells [58]. Studies have shown that pathological conditions such as diabetes, hyperglycemia, and chronic inflammation result in mitochondrial respiratory chain dysfunction, which in turn leads to excessive ROS production, causing damage to lipids, proteins, and DNA, and ultimately endothelial cell dysfunction or even death [59,60]. Leveraging the oxygen vacancies on their surface and the rapid, reversible switching of Ce^3+^/Ce^4+^, CeO_2_ NPs continuously quenched multiple free radicals, protected membrane integrity, and preserved the functional activity of endothelial cells, key effectors essential for subsequent angiogenesis.

Second, CeO_2_ NPs effectively promote angiogenesis through a mechanism centered on stabilizing HIF-1α and upregulating VEGF expression. During the proliferative phase of wound healing, the formation of new blood vessels is critical for the delivery of oxygen and nutrients [61]. Studies have shown that CeO_2_ NPs can endogenously stabilize HIF-1α by modulating the intracellular oxygen environment, thereby preventing its rapid degradation [62]. Stabilized HIF-1α subsequently translocates to the nucleus, where it functions as a transcription factor, initiating the expression of a range of proangiogenic genes, including VEGF. Research on cerium-containing bioactive glass further supports this mechanism, as its extract promotes the proliferation, migration, and tubule formation of lymphatic endothelial cells via the HIF-1α/VEGFR-3 pathway, providing additional evidence for cerium’s critical role in activating this pathway [63]. The resulting VEGF secretion stimulates endothelial cell proliferation and migration, ultimately leading to the formation of new capillary networks that supply adequate oxygen and nutrients to regenerating tissues.

Finally, CeO_2_ NPs can modulate the immune microenvironment by orchestrating macrophage polarization from the proinflammatory M_1_ to the reparative M_2_ phenotype, thereby indirectly promoting angiogenesis and tissue repair. During normal wound healing, macrophages undergo a timely transition from the M_1_ phenotype during the early inflammatory phase to the M_2_ phenotype in the proliferative phase, accompanied by the secretion of pro-reparative factors such as TGF-β and platelet-derived growth factor (PDGF) [64]. However, in chronic nonhealing wounds, such as diabetic ulcers, this phenotypic transition is impaired, leading to sustained M_1_-dominant inflammation. Studies have demonstrated that functionalized CeO_2_ NPs, including those modified with dextran, promote macrophage polarization toward the M_2_ phenotype [65]. These M_2_-polarized macrophages subsequently secrete a range of proangiogenic and profibrogenic cytokines, including TGF-β and PDGF, which not only directly support angiogenesis but also stimulate fibroblast proliferation and collagen deposition, thereby synergistically accelerating the wound healing process [6]. This strategy of creating a pro-regenerative microenvironment by modulating immune cell function offers a novel approach to treating inflammatory skin diseases.

## 4. Applications in Different Inflammatory Skin Disorders

### 4.1. Atopic Dermatitis

AD is a chronic, relapsing inflammatory skin disorder driven by genetic susceptibility, immune dysregulation, and environmental factors. Its pathophysiology is defined by a triad of Th2-skewed immune polarization, skin barrier dysfunction, and intractable pruritus-induced scratching [66]. These components form a self-reinforcing loop: Th2 cytokines (e.g., IL-4 and IL-13) downregulate barrier molecules, such as filaggrin, in keratinocytes [67]; barrier disruption facilitates the invasion of allergens and microbes, further activating antigen-presenting cells and sustaining Th2 responses [67,68]. Concurrently, severe itching induces scratching, and the resulting mechanical injury triggers the release of ROS, which serve as both damage markers and proinflammatory second messengers, amplifying local inflammation via pathways such as NF-κB [67,68]. Such establishes a positive “itch–scratch–inflammation” feedback loop. Such pathological complexity limits the efficacy of single-target interventions, underscoring the need for therapeutic platforms that can simultaneously target multiple nodes. In this context, CeO_2_ NPs offer unique potential through sustained free radical scavenging and self-regenerating catalytic activity. By leveraging these properties, CeO_2_ NP-based strategies can modulate oxidative stress and support skin barrier function within the AD microenvironment.

First, at the level of oxidative stress, CeO_2_ NPs can precisely interrupt the inflammation amplification loop induced by itching and scratching. Mechanical injury resulting from scratching leads to a sharp increase in local ROS levels. These ROS not only directly damage cellular structures but also, more importantly, function as signaling molecules that activate inflammatory pathways such as NF-κB and mitogen-activated protein kinase (MAPK), promoting the release of proinflammatory mediators, including thymic stromal lymphopoietin and TNF-α, thereby converting mechanical stimuli into sustained inflammatory signals [69]. By leveraging their SOD- and CAT-mimetic activities, CeO_2_ NPs efficiently scavenge excess ROS generated in skin tissue, thereby blocking the activation of inflammatory pathways by oxidative stress [70]. This intervention effectively interrupted the “scratching–oxidative stress–inflammation” positive feedback loop, thereby suppressing the persistent amplification of local inflammatory responses.

Second, at the level of barrier repair, CeO_2_ NPs synergistically promote structural and functional restoration of the skin barrier through a dual mechanism involving direct antioxidant protection and indirect anti-inflammatory regulation. At the direct level, by leveraging their exceptional ROS-scavenging capacity, CeO_2_ NPs effectively reduce oxidative stress within keratinocytes, thereby mitigating oxidative interference with normal cellular differentiation and intercellular lipid synthesis [71,72]. At the indirect level, these nanoparticles sustain keratinocyte function and viability by inhibiting inflammatory signaling, thereby facilitating skin barrier formation [73]. Recent studies have confirmed that porous cerium oxide nanorods effectively modulate the interaction between keratinocytes and macrophages within the inflammatory microenvironment characteristic of AD, thereby reducing levels of inflammatory cytokines and promoting the expression of barrier-related molecules [5]. This nanozyme-based strategy, centered on ROS scavenging, has been demonstrated to significantly reduce epidermal thickness and immune marker levels in mouse models of AD. The convergent actions of these mechanisms collectively facilitate the restoration of stratum corneum integrity and function, reduce transepidermal water loss, and reinforce the skin’s barrier capacity against exogenous insults.

It is important to clarify that AD is a chronic inflammatory disease driven by a complex interplay of multiple pathological factors, including immune dysregulation, barrier dysfunction, and oxidative stress. Given the intricate nature of its pathogenesis network, single-target interventions often fail to achieve optimal therapeutic outcomes. Current treatment guidelines recommend the use of combination therapies for patients with moderate-to-severe AD. Although CeO_2_ NPs effectively alleviate oxidative stress and modulate the inflammatory microenvironment, their mechanism of action primarily involves downstream effects on inflammation and oxidative damage [5], with no evidence to date demonstrating their ability to specifically block upstream signaling of Th2-type core pathogenic mediators such as IL-4 and IL-13. Therefore, a more appropriate role for CeO_2_ NPs in AD therapy is as a multifunctional adjuvant nanoplatform. Leveraging their multitarget mode of action, which encompasses antioxidant, immunomodulatory, and barrier-protective functions, these nanoparticles can synergize with conventional treatments. Moreover, they hold promise as drug delivery vehicles for combination with corticosteroids [74], Janus kinase (JAK) inhibitors [75], or biologics [76], potentially reducing the required dosage and associated side effects of primary agents while achieving broader therapeutic coverage of the complex AD pathological network.

### 4.2. Psoriasis

Psoriasis is a chronic, recurrent, immune-mediated inflammatory skin disease driven by the interplay of genetic susceptibility and environmental factors, with a global prevalence of approximately 1–3%. Its impact on patients’ quality of life is comparable to that of cancer or diabetes [77]. The pathogenesis of psoriasis centers on dysregulation of both innate and adaptive immunity, with particular emphasis on the aberrant activation of the IL-23/Th17 axis as a critical nexus. Activated dendritic cells secrete IL-23, which induces Th17 cell differentiation and subsequent release of effector cytokines, including IL-17 and IL-22 [78]. IL-17, in turn, acts on keratinocytes, driving their abnormal proliferation and activation and inducing the secretion of chemokines that recruit neutrophils and T cells to lesion sites, thereby establishing a self-perpetuating inflammatory amplification loop [78]. Histologically, this immune cascade manifests as epidermal hyperkeratosis, parakeratosis, acanthosis, and pronounced dermal infiltration of inflammatory cells [79]. Consequently, effective interventions targeting the IL-23/Th17 axis, inhibiting aberrant keratinocyte proliferation, and blocking downstream inflammatory signaling have emerged as key therapeutic strategies for psoriasis.

In the pathological progression of psoriasis, oxidative stress and immune inflammation form a mutually reinforcing vicious cycle. ROS levels are markedly elevated in lesional skin, functioning not only as products of the inflammatory response but also as key mediators that drive aberrant keratinocyte proliferation and sustained inflammation. CeO_2_ NPs, by virtue of their unique redox activity, efficiently scavenge superoxide anions and hydrogen peroxide, thereby protecting keratinocytes from oxidative damage [80]. More importantly, these nanoparticles not only exhibit broad-spectrum antioxidant effects but also modulate key inflammatory pathways, thereby intervening in the immunopathological processes of psoriasis. In an imiquimod-induced mouse model of psoriasis, treatment with mitochondrion-targeted CeO_2_ NPs in combination with all-trans retinoic acid resulted in significantly reduced levels of IL-6 and TNF-α in lesional tissues [81]. In another study, local or systemic administration of CeO_2_ NPs markedly alleviated splenomegaly, reduced Psoriasis Area and Severity Index scores, and decreased lipid peroxidation levels [82]. Mechanistic investigations further revealed that CeO_2_ NPs significantly downregulate the expression of Th cell-mediated cytokines associated with the IL-17/IL-23 axis, including IL-17, IL-22, and IL-23, while also inhibiting the activation of key inflammatory mediators such as NF-κB and cyclooxygenase-2 [82], suggesting that these compounds have the potential to intervene in the core immune pathways underlying psoriasis.

Although existing studies have demonstrated that CeO_2_ NPs can ameliorate pathological changes, such as epidermal hyperplasia, by scavenging excess ROS, disrupting the vicious “oxidative stress-inflammation” cycle, and effectively downregulating key inflammatory cytokines in the Th17 axis, significant gaps remain in understanding the underlying mechanisms. Current evidence predominantly focuses on broad-spectrum antioxidant effects and downstream regulation of inflammatory mediators, with limited direct evidence demonstrating that CeO_2_ NPs act on key upstream molecules of the IL-23/Th17 axis, such as signals involved in dendritic cell activation or T-cell differentiation. Consequently, the mechanistic description remains relatively superficial. Furthermore, the fate of CeO_2_ NPs within the complex lesional microenvironment, including their cellular uptake pathways, intracellular localization, dynamic valence-state changes, and long-term biological effects, has yet to be systematically elucidated. These gaps not only constrain a precise understanding of their therapeutic mechanisms but also pose challenges to achieving targeted regulation of efficacy and evaluating long-term safety. Therefore, future research should move beyond a phenomenological description toward mechanistic elucidation, focusing on the direct interactions between CeO_2_ NPs and key immune cells as well as critical signaling nodes, thereby facilitating the effective translation of CeO_2_ NPs into clinical therapies for psoriasis.

### 4.3. Diabetic Wounds

The development and progression of chronic nonhealing wounds, such as diabetic ulcers, arise from a complex pathological process driven by multiple interrelated factors. Under persistent stimulation from the hyperglycemic microenvironment, mitochondrial electron transport chain dysfunction leads to excessive ROS production, triggering severe oxidative stress and DNA damage [83,84]. Accumulated ROS not only directly disrupt cellular structures but also, more critically, function as key second messengers that activate inflammatory pathways such as NF-κB, resulting in substantial release of proinflammatory cytokines, including TNF-α and IL-6, thereby establishing a persistent inflammatory state dominated by M_1_-type macrophages. Concurrently, the hyperglycemic environment provides fertile ground for bacterial colonization and biofilm formation [85,86], with subsequent infection further exacerbating local inflammation. This vicious cycle of oxidative stress and inflammation directly impairs endothelial cell function, compromising angiogenesis and preventing the wound from progressing beyond the inflammatory phase into the proliferative and remodeling phases [87,88]. In addressing this complex pathological network, CeO_2_ NPs, owing to their unique reversible valence-state-switching capability, enable systematic intervention in wound healing via synergistic, multitarget mechanisms.

At the level of oxidative stress, CeO_2_ NPs scavenge excess ROS in the wound microenvironment by decomposing superoxide anions and hydrogen peroxide. Studies have shown that functionalized CeO_2_ NPs significantly reduce ROS levels in diabetic wounds, restore intracellular redox homeostasis, facilitate fibroblast migration, and promote endothelial cell tube formation, thereby establishing a foundation for subsequent tissue repair [89]. In terms of inflammatory regulation and immune remodeling, CeO_2_ NPs attenuate inflammatory responses by scavenging ROS, acting as upstream signaling molecules, and modulating the NF-κB signaling pathway [90]. More critically, CeO_2_ NPs induce macrophage polarization from the proinflammatory M_1_ phenotype toward the proreparative M_2_ phenotype, effectively disrupting the “inflammation–oxidative stress” positive feedback loop through immunomodulation [91]. This dual regulatory mechanism, encompassing both the suppression of signaling pathways and the reshaping of immune cell populations, creates a favorable microenvironment that enables the wound to transition into the proliferative phase. Regarding their anti-infective and pro-regenerative effects, CeO_2_ NPs exhibit antibiotic-independent antimicrobial mechanisms. Functionally modified CeO_2_ NPs act on bacterial membranes via electrostatic interactions, compromising membrane integrity and penetrating biofilm matrices, thereby demonstrating significant antimicrobial and antibiofilm efficacy against clinically relevant strains such as Pseudomonas aeruginosa [49]. Concurrently, by scavenging ROS and protecting endothelial cell function, CeO_2_ NPs enhance migration, tube formation, and the expression of angiogenesis-related genes in human umbilical vein endothelial cells, underscoring their potential to promote angiogenesis [92].

In summary, CeO_2_ NPs exert synergistic effects across multiple critical stages of wound healing through a cascade mechanism involving ROS scavenging, modulation of inflammation, antimicrobial activity, and pro-angiogenic effects. This multitarget intervention paradigm, integrating antioxidant, immunomodulatory, antimicrobial, and pro-regenerative functions, offers a pathogenesis-driven, integrated strategy for treating chronic nonhealing wounds, such as diabetic ulcers. However, challenges remain regarding the insufficient responsiveness of CeO_2_ NPs to the pathophysiological features of diabetic wounds, including hyperglycemia, low pH, and elevated ROS levels, which can lead to premature burst release or functional inactivation [93]. A promising direction for future development is to combine CeO_2_ NPs with glucose oxidase to leverage cascade catalytic reactions that deplete local glucose, alleviate hypoxia, and concurrently enhance antimicrobial efficacy.

### 4.4. Skin Photoaging

Skin photoaging refers to premature skin aging resulting from chronic exposure to UV radiation. Its core pathological features include UV-induced burst production of ROS, which in turn triggers oxidative stress, inflammatory responses, and degradation of the extracellular matrix, characterized by collagen fragmentation and aberrant crosslinking, as well as elastosis [94]. These cumulative effects ultimately include skin laxity, wrinkle formation, and hyperpigmentation. Owing to their unique redox properties, CeO_2_ NPs exhibit significant therapeutic potential for antiphotoaging via distinct mechanisms.

First, the antiphotoaging efficacy of CeO_2_ NPs is fundamentally attributed to their reversible redox activity, conferred by the coexistence of surface Ce^3+^ and Ce^4+^, enabling direct scavenging of excessive UV-induced ROS. Studies have shown that glycerol-modified ultrasmall CeO_2_ NPs significantly increase the surface Ce^3+^ content and oxygen vacancy concentration, thereby enhancing their ability to scavenge 2,2-diphenyl-1-picrylhydrazyl radicals, superoxide anions, and hydroxyl radicals [95]. In UV-irradiated human keratinocyte models, glycerol-modified CeO_2_ NPs effectively inhibited intracellular ROS production, providing superior photoprotective effects at safe concentrations [95]. At the level of apoptosis, CeO_2_ NPs significantly reduce UV-induced apoptosis by decreasing caspase-3/7 activity and preventing mitochondrial membrane potential loss [96]. In terms of cell viability and proliferation, fibroblasts treated with CeO_2_ NPs exhibit increased survival, proliferative capacity, and migratory ability following UV irradiation [71].

Second, ultraviolet-induced ROS can function as second messengers to activate signaling pathways, such as c-Jun N-terminal kinase (JNK), leading to upregulated expression of matrix metalloproteinases and, consequently, excessive collagen degradation [97], a primary contributor to wrinkle formation and skin laxity in photoaged skin. Studies have demonstrated that CeO_2_ NPs significantly inhibit matrix metalloproteinase-2 expression and secretion in fibroblasts following UV irradiation by modulating the JNK signal transduction pathway [71].

Finally, inducing collagen synthesis or supplementing collagen in the skin represents a primary strategy for addressing skin photoaging [98]. It has been reported that, when combined with collagen gel, CeO_2_ NPs inhibit collagen degradation, reduce levels of aging-related biomarkers, and increase collagen deposition, with antiaging effects persisting for up to 7 months [99].

Beyond CeO_2_, other cerium-containing materials have also attracted attention as potential photoprotective agents. In particular, cerium phosphate (CePO_4_) exhibits strong UV absorption together with low photocatalytic activity, making it a promising candidate for inorganic UV-filter applications [100]. CePO_4_-containing sunscreen formulations have also demonstrated favorable physicochemical stability and relatively low interaction with conventional organic UV filters, suggesting potential formulation advantages over commonly used inorganic filters [101]. More recent studies have further shown that the photoprotective properties of cerium phosphates depend on their composition and cerium oxidation state. Notably, selected Ce (IV) phosphate materials exhibit UV-shielding activity comparable to nanocrystalline CeO_2_ while maintaining low photocatalytic activity and favorable cytocompatibility [102]. These findings indicate that cerium phosphates may complement CeO_2_-based strategies by providing an alternative route to UV attenuation with limited photocatalytic ROS generation. Nevertheless, their long-term cutaneous safety, skin retention, and performance in clinically relevant sunscreen formulations require further investigation before translation.

## 5. Advanced Functionalization and Delivery Strategies Based on CeO_2_ NPs

### 5.1. Structural Functionalization

The biological efficacy of CeO_2_ NPs originates from their distinctive crystal structure and the consequent reversible Ce^3+^/Ce^4+^ redox cycling. By precisely modulating their intrinsic physicochemical properties, the antioxidant and catalytic performance of these nanoparticles can be substantially improved, laying the groundwork for biomedical applications. Current strategies for structural optimization focus primarily on three principal avenues: doping modification, morphological control, and defect engineering.

Doping modification represents a key strategy for tuning the physicochemical and catalytic properties of CeO_2_ NPs. Introducing heterovalent metal ions (such as Fe^3+^, Zn^2+^, Cu^2+^, and other aliovalent dopants) into the CeO_2_ lattice can modify its local coordination environment, electronic structure, Ce^3+^/Ce^4+^ balance, and oxygen-vacancy formation. Transition-metal doping can thereby alter the reactivity of lattice oxygen and catalytic behavior. For example, Mn and Cr dopants have been reported to lower the oxygen-vacancy formation energy of ceria and enhance catalytic activity in oxidation reactions [103]. Rare-earth-ion doping provides an additional means of regulating ceria defect chemistry. In particular, acceptor doping with Gd^3+^ promotes charge-compensating oxygen-vacancy formation, thereby altering the oxygen nonstoichiometry and defect structure of CeO_2_ and modifying its physicochemical properties [23]. Importantly, however, increasing oxygen-vacancy concentration does not necessarily translate into proportionally enhanced biological antioxidant activity. In Gd-doped CeO_2_ NPs, increasing the doping level altered the Ce^3+^/Ce^4+^ state and decreased antioxidant potential without producing a corresponding increase in acute toxicity under the experimental conditions examined [24]. These findings indicate that the biological redox behavior of doped CeO_2_ depends not simply on the abundance of oxygen vacancies or a single cerium valence state, but also on the specific defect configuration and the capacity for reversible Ce^3+^/Ce^4+^ cycling. Other rare-earth-based strategies may further modify catalytic stability; for instance, Yb-assisted stabilization of Pd within ceria has been reported to suppress Pd aggregation under reducing conditions and sustain catalytic activity [104]. Collectively, these findings demonstrate that ion doping can impart distinct physicochemical properties to CeO_2_, although the resulting biological performance must be evaluated for each specific dopant and material configuration.

Nevertheless, the performance gains achieved through metal doping should be balanced against dopant-specific safety considerations. The biological effects of doped CeO_2_ NPs depend not only on changes in the defect chemistry of the ceria matrix but also on the identity and concentration of the incorporated metal. For example, comparative studies in human HaCaT keratinocytes demonstrated that Cr- and Co-doped CeO_2_ NPs produced greater dose- and time-dependent cytotoxicity than undoped CeO_2_, whereas Fe-, Mn-, and Ni-doped counterparts did not produce comparable increases in toxicity under the same experimental conditions [105]. These findings highlight that apparently similar doping strategies can result in substantially different biological responses. From a translational perspective, the physicochemical stability of the doped material also requires careful consideration. Changes in nanoparticle structure or partial dissolution under biological conditions could potentially affect dopant retention and metal-ion release, introducing safety considerations distinct from those of the CeO_2_ matrix itself. Therefore, optimization of doped CeO_2_ NPs should not focus solely on maximizing oxygen-vacancy concentration or catalytic performance. Future studies should systematically characterize dopant incorporation, oxidation state, phase segregation, and lattice stability; quantify dopant and cerium-ion release under physiologically and pathologically relevant conditions; and evaluate dose-, time-, and cell-type-dependent toxicity, particularly following repeated or prolonged cutaneous exposure. Such integrated risk-benefit assessment will be essential for determining whether the functional advantages conferred by a given dopant justify the additional material-specific safety considerations.

Morphology control represents another important strategy for tuning the catalytic and biological properties of CeO_2_ NPs. Nanoparticle morphologies, such as nano-octahedra, nanorods, and nanocubes, directly influence the exposed crystal facets, oxygen vacancy concentration, and surface energy, which in turn affect their biological behavior and catalytic performance [106]. Studies have demonstrated that variations in surface atomic arrangement and electronic structure across different crystal facets lead to significant differences in oxygen vacancy formation energy and reactivity. For instance, nanorods with exposed high-energy facets exhibit substantially higher surface Ce^3+^ concentrations and oxygen vacancy densities than nanooctahedra with low-energy facets, due to their higher surface energy and lower oxygen vacancy formation energy [107]. This facet-dependent activity pattern underpins superior free-radical scavenging capacity, antimicrobial activity, and catalytic performance, providing critical guidance for the rational design of high-performance CeO_2_ NPs. To achieve precise control of morphology, various strategies have been developed. For instance, introducing acetate ions as surface modifiers during synthesis exploits their selective adsorption on specific crystal facets, effectively driving the morphological transition of nanoparticles from truncated octahedra to cubes, with pH and acetate concentration serving as key control factors [108]. Additionally, optimizing deposition conditions, such as reagent mixing methods and drying temperatures, is critical for obtaining nanoparticles with uniform size and good dispersibility [109]. Thus, through morphology control, the exposed crystal facets, surface defects, and electronic structure of CeO_2_ NPs can be systematically tuned, thereby enabling performance optimization across catalysis, energy, and biomedicine applications.

Defect engineering, particularly the regulation of oxygen vacancies, lies at the core of optimizing the performance of CeO_2_ NPs. As the most active defect sites on the ceria surface, oxygen vacancies not only underpin the presence of Ce^3+^ but also function as active centers for catalytic reactions. Studies have shown that oxygen vacancy concentrations can be effectively modulated by controlling synthesis conditions, such as the calcination temperature, or by employing green synthesis approaches. For instance, CeO_2_ NPs synthesized using polyphenol-rich plant extracts, including coffee husk extract, exhibit abundant surface Ce^3+^ and oxygen vacancies [110]. However, the subsequent calcination treatment, while enhancing crystallinity, reduces oxygen vacancies and lattice strain, thereby decreasing antioxidant activity [110,111]. Therefore, rational optimization of oxygen-vacancy concentration, while maintaining nanoparticle stability, represents a critical factor in achieving efficient catalytic redox reactions.

### 5.2. Biomimetic Surface Modification

Cell membrane-coated inorganic nanoparticles have garnered attention as promising intelligent delivery platforms with substantial therapeutic potential [112]. The core principle of cell membrane coating technology is to leverage the complex composition and biological functions of natural cell membranes to functionalize synthetic nanoparticles, thereby generating “nanoscale cellular mimics.” These nanoscale mimics retain selected intrinsic properties of the source cells, conferring distinctive characteristics, including prolonged circulation, immune evasion, and targeted localization to sites of inflammation.

Cell membranes derived from different sources possess distinct characteristics. Table 2 summarizes the features of selected cell membrane-coated nanoparticles in various disease contexts. This biomimetic strategy, encapsulating inorganic nanoparticles within natural cell membranes, produces constructs with excellent biocompatibility, prolonged circulation time, and potential for active targeting, thereby offering a promising approach for the precise, efficient, and low-toxicity treatment of inflammatory skin diseases. For instance, Zeng and colleagues developed a biomimetic macrophage membrane-coated nanoparticle system that leverages receptors such as TNFR1 and IL-6R expressed on the membrane surface to neutralize inflammatory cytokines while delivering the neddylation inhibitor MLN4924 in a targeted manner. This dual immunomodulatory strategy inhibits M_1_ macrophage polarization and promotes vascular endothelial cell function [113]. Furthermore, engineered cells can confer active immunomodulatory functions to their membranes. For example, macrophage membranes preconditioned with interferon-gamma exhibit significantly upregulated expression of programmed death-ligand 1 (PD-L1) and various proinflammatory cytokine receptors upon stimulation. Such membranes can suppress T-cell activation and Th17 differentiation via the PD-1/PD-L1 axis, thereby remodeling the immune microenvironment in psoriatic lesions and restoring the Th17/Treg immune balance [114].

Furthermore, metabolic glycoengineering enables the introduction of azide groups (-N_3_) onto the cell membrane, which, in combination with pre-installed dibenzocyclooctyne (DBCO) groups at the lesion site, facilitates highly efficient, specific covalent binding via click chemistry, thereby achieving high-selectivity enrichment at inflamed sites. Wang et al. incorporated DBCO groups onto the surface of keratinocytes and immune cells within psoriatic lesions via metabolic glycoengineering [126]. Concurrently, they constructed N_3_-labeled HEK-293T cell membrane vesicles encapsulating IR-780-PLGA nanoparticles (N_3_-NV-INPs), enabling specific targeted delivery to the lesion site through bioorthogonal click chemistry. Upon near-infrared irradiation, the N_3_-NV-INPs exerted synergistic photodynamic and photothermal effects mediated by IR-780, effectively inhibiting excessive keratinocyte proliferation, reducing local immune cell infiltration, and significantly downregulating the expression levels of key inflammatory cytokines, including IL-6, IL-17, and TNF-α. This strategy elegantly circumvents the complexities associated with traditional antibody–antigen targeting, enabling highly selective drug delivery.

Despite the relatively underexplored application of cell membrane coating technology to CeO_2_ NPs, the demonstrated advantages of the aforementioned inorganic nanoplatforms, particularly in biomimetic camouflage, precise targeting, and multimodal therapy, support the reasonable inference that this strategy offers a promising avenue to address key bottlenecks in the use of CeO_2_ NPs for treating inflammatory skin diseases. Specifically, it has the potential to overcome longstanding challenges, including insufficient targeting capability, suboptimal accumulation at inflammatory sites, and the risk of nonspecific uptake. The diversity of cell membrane sources provides opportunities to expand the functional repertoire of CeO_2_ NPs. For instance, coating with activated immune cell membranes may confer active chemotactic homing to inflamed sites, synergizing with the intrinsic ROS-scavenging activity of cerium oxide to modulate excessive immune responses [127], while red blood cell membranes can further optimize biocompatibility and circulation stability [128]. Thus, cell membrane coating technology not only compensates for the limitations of CeO_2_ NPs in targeted delivery and in vivo stability but also enables multilevel synergy encompassing targeting, antioxidant activity, and immunomodulation through functionalization of the membrane surface. The deep integration of these two approaches holds significant promise for developing intelligent nanoplatforms that combine precise inflammatory targeting, controlled local retention, and potent antioxidant therapy, offering new directions for precise intervention in inflammatory skin disorders.

### 5.3. Formulation Hybridization

**Figure 3:**
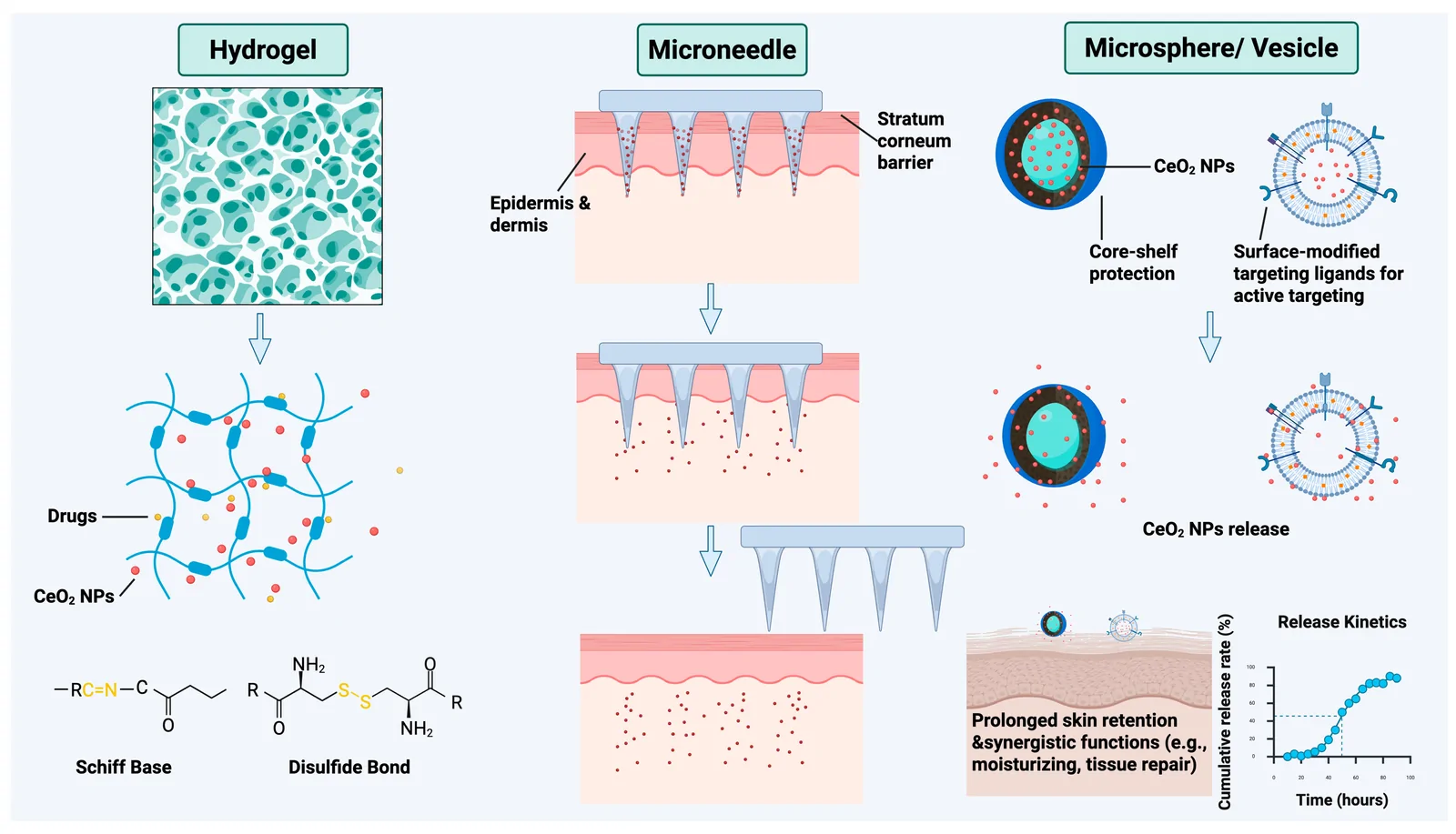
Schematic diagram of skin delivery strategies and functions of CeO_2_ NPs. Created in BioRender. Liu, W. (2026) https://BioRender.com/nt62yvx (accessed on 13 September 2026).

Although CeO_2_ NPs exhibit considerable potential for modulating the inflammatory skin microenvironment owing to their distinctive multienzyme activities, their clinical translation remains limited by key bottlenecks, including limited penetration into viable skin layers, insufficient retention time at the lesion site, and a lack of synergistic therapeutic modalities. In recent years, advances in biomedical materials, such as hydrogels, microneedles, and microspheres, have facilitated the application of CeO_2_ NPs in the treatment of inflammatory skin diseases (Figure 3). By integrating CeO_2_ nanozymes with advanced biomaterials [6], innovative formulation strategies have enabled a paradigm shift from a purely antioxidant approach toward a comprehensive framework that encompasses controlled release, enhanced retention, and synergistic therapy, thereby offering efficient and innovative interventions for inflammatory skin diseases.

Hydrogels, characterized by their three-dimensional porous networks and high-water content, can mimic the native extracellular matrix. They provide a sustained moist environment at the wound site, thereby facilitating cell migration and nutrient exchange while effectively absorbing exudate and reducing the risk of infection [129]. Building on these features, researchers have further endowed hydrogels with the ability to release in response to pathological microenvironmental cues, representing a critical advancement for enhancing therapeutic precision. By incorporating dynamic chemical bonds, such as Schiff base [130] and disulfide [131] bonds, hydrogel systems capable of specific degradation under conditions of high oxidative stress, acidity, or hyperglycemia have been developed, enabling on-demand delivery of CeO_2_ NPs and other active components. For instance, dynamically crosslinked hydrogels containing disulfide bonds can modulate their structure in response to oxidative environments, thereby increasing local accumulation and sustained action of CeO_2_ NPs [130]. Such intelligently responsive properties allow hydrogels to dynamically adapt to evolving pathological changes across different stages of wound healing, thereby improving treatment precision. Furthermore, the use of composite platforms that integrate CeO_2_ NPs with hydrogels enables synergistic therapy involving multiple mechanisms. By coloading antimicrobial agents, naturally derived active ingredients, or photothermal agents, these platforms achieve coordinated antibacterial, anti-inflammatory, pro-angiogenic, and anti-glycation effects on the foundation of antioxidant activity, offering particular promise for chronic nonhealing wounds such as diabetic foot ulcers and infected wounds [130,131,132,133].

Microneedle delivery systems offer an effective strategy to overcome the stratum corneum barrier for the intradermal delivery of cerium oxide nanozymes. Leveraging microneedle penetration, this system not only enhances the bioavailability of nanozymes in the skin but also facilitates synergistic therapy by co-loading with therapeutic agents. For example, in the treatment of psoriasis, researchers have developed a ROS-responsive bilayer microneedle system in which tips are loaded with methotrexate for targeted release via responsive materials, while the backing layer encapsulates CeO_2_ NPs to provide sustained anti-inflammatory and antioxidant effects, thereby effectively interrupting the pathological cycle [70]. Furthermore, under conditions such as chronic diabetic wounds and irritant contact dermatitis, multifunctional cerium oxide microneedle systems incorporating antimicrobial ions and antibiotics integrate multiple functions, including antimicrobial, antioxidant, anti-inflammatory, and angiogenesis, to comprehensively remodel the diseased microenvironment and significantly increase the repair efficiency of chronic nonhealing wounds [9,134].

Research efforts on microsphere- and vesicle-based formulations have primarily focused on encapsulating CeO_2_ NPs within liposomes [135], polymersomes [136], or biodegradable microspheres [137] to achieve protective delivery, sustained release, and targeted functionalization. By encapsulating cerium oxide nanozymes within these carriers, premature inactivation within complex physiological environments can be effectively prevented, thereby enhancing cutaneous delivery and improving local retention [81]. Furthermore, surface modification with targeting ligands, such as amino functionalization [138], enables active targeting to the lesion site. For example, CeO_2_-loaded microspheres using hydroxypropyl cellulose as a matrix can efficiently scavenge ROS by leveraging the multienzyme-like activities of the nanozymes, significantly alleviating skin inflammation and immune activation in a mouse model of psoriasis [137]. More importantly, CeO_2_-based functionalized microspheres can be combined with other microspheres with moisturizing or pro-reparative properties to form multicomponent, synergistic therapeutic strategies, thereby offering a more comprehensive approach to intervening in the multifactorial pathogenic networks underlying inflammatory skin diseases.

In summary, through the innovative design of microneedles, hydrogels, and microsphere-based systems (including liposomes and vesicles), CeO_2_ NPs have enabled the development of a comprehensive therapeutic strategy for inflammatory skin disorders centered on three core pillars: enhanced transdermal delivery, intelligent microenvironment-responsive release, and synergistic multi-mechanistic action. This approach leverages the distinct structural advantages of each formulation: hydrogels provide a hydrated scaffold and enable responsive release; microneedles bypass the skin barrier to achieve precise delivery; and microspheres and vesicles preserve nanozyme stability while facilitating targeted localization and harnessing the multienzyme activity and broad-spectrum regulatory capacity of CeO_2_ NPs. Collectively, these attributes establish a versatile and promising therapeutic platform for complex inflammatory skin conditions, including chronic nonhealing wounds, psoriasis, alopecia, and infected wounds.

### 5.4. Cutaneous Fate, Biotransformation, and Safety Considerations

The therapeutic performance and safety of topically administered CeO_2_ NPs depend not only on their intrinsic catalytic properties and delivery efficiency but also on their subsequent penetration, localization, transformation, and persistence within the skin. The stratum corneum constitutes a major barrier to nanoparticle entry, and available evidence suggests that CeO_2_ NPs exhibit only limited permeation across intact human skin. In an ex vivo Franz diffusion-cell study using 17 nm CeO_2_ NPs, transdermal permeation after 24 h was very low, whereas disruption of the skin barrier substantially increased cutaneous uptake and cerium deposition within the dermis [139]. This distinction is particularly relevant to inflammatory skin diseases and chronic wounds, in which barrier integrity is frequently compromised. Accordingly, penetration behavior observed in healthy intact skin may not fully predict nanoparticle distribution under pathological conditions. Cutaneous localization is also likely to depend on particle size, surface chemistry, aggregation state, and formulation, while delivery systems such as microneedles can intentionally bypass the stratum corneum and increase deposition within viable skin layers. However, the long-term retention, repeated-dose accumulation, clearance kinetics, and potential systemic exposure of CeO_2_ NPs following topical administration remain insufficiently characterized. Future studies should therefore quantify the distribution of both particulate and total cerium across the stratum corneum, viable epidermis, dermis, and appendageal compartments under both intact and disease-relevant barrier conditions.

Once deposited within biological tissues or internalized by cells, CeO_2_ NPs should not be regarded as chemically inert entities with permanently fixed physicochemical properties. Their fate can be influenced by local pH, redox conditions, complexing ligands, phosphate availability, nanoparticle size and surface chemistry, and intracellular compartmentalization. Experimental studies indicate that CeO_2_ dissolution is strongly environment-dependent and is generally limited under neutral conditions, whereas acidic conditions and selected complexing or reducing agents can promote partial dissolution and the release of soluble cerium species [140]. Phosphate can strongly interact with the CeO_2_ surface and, depending on the surrounding chemical environment, influence dissolution and subsequent transformation; for example, phosphate adsorption has been shown to suppress CeO_2_ dissolution under some aqueous conditions [140], whereas phosphate-containing environments can support the formation of cerium phosphate following appropriate dissolution and redox processes [141]. Importantly, intracellular studies provide direct evidence that internalized nanoceria can undergo partial dissolution and subsequent biotransformation. In human and murine macrophages, partially dissolved CeO_2_ nanoparticles have been observed together with newly formed crystalline CePO_4_ structures, indicating redistribution of cerium species and reprecipitation within the intracellular environment [142]. Such transformations may modify surface composition, cerium oxidation state, and redox activity and therefore alter the biological behavior of the original nanomaterial [143]. These observations do not imply rapid or complete dissolution of CeO_2_ under physiological conditions; rather, they demonstrate that nanoceria can undergo condition-dependent and potentially prolonged physicochemical transformation. Characterization of dissolution kinetics, cerium speciation, surface-valence changes, and transformation products under skin-relevant extracellular and intracellular conditions is therefore essential for predicting both therapeutic persistence and long-term safety. Studies of doped CeO_2_ should additionally determine whether such transformations affect dopant retention or promote the release of incorporated metal species.

These considerations also underscore the need for a more systematic assessment of topical toxicology. Although multiple in vitro and preclinical studies have reported favorable cytocompatibility and protective or pro-reparative effects of appropriately formulated CeO_2_ NPs in keratinocytes, fibroblasts, and wound-healing models, biological responses are not uniform across nanoparticle formulations or exposure conditions [144]. However, the available evidence remains predominantly preclinical and does not constitute a comprehensive dermatological safety assessment. Indeed, the biological response to CeO_2_ NPs can vary with particle size, morphology, surface chemistry, concentration, exposure duration, and incorporated dopants; for example, transition-metal doping has been shown to alter CeO_2_-associated cytotoxicity in human keratinocytes in a dopant-dependent manner [105]. Therefore, short-term cell viability alone is insufficient to establish cutaneous safety. Future evaluations should include dose- and time-dependent effects on keratinocytes, fibroblasts, and resident immune cells; irritation and sensitization potential; inflammatory and oxidative responses; genotoxicity where relevant; barrier integrity; and histopathological changes following repeated topical exposure. Particular attention should be paid to diseased or wounded skin, where increased penetration may expose viable epidermal and dermal cells to nanoparticle concentrations that differ substantially from those encountered after application to intact skin. Long-term studies should further distinguish desirable local therapeutic retention from undesirable persistent accumulation and assess whether retained CeO_2_, released cerium species, or transformation products produce delayed local or systemic effects. Collectively, integration of penetration, biotransformation, clearance, and repeated-dose toxicology will be necessary to define an exposure window in which the local therapeutic benefits of CeO_2_ NPs can be achieved without unacceptable long-term cutaneous or systemic risk.

## 6. Conclusions and Outlook

Owing to their reversible redox cycling between Ce^3+^ and Ce^4+^, multienzyme-mimetic activities, and physicochemical tunability, CeO_2_ NPs have emerged as promising candidates for multifunctional intervention in inflammatory skin diseases. Their catalytic and biological activities can be modulated by multiple structural parameters, including particle size, morphology, crystal facet, doping, and surface modification. For example, reducing particle size can increase the specific surface area and enhance surface Ce^3+^ exposure of redox-active sites, crystal-facet engineering can influence oxygen-vacancy distribution, and heterovalent ion doping can further modify defect chemistry and redox behavior. Building on these intrinsic properties, surface engineering and biomimetic modifications can improve dispersibility, biocompatibility, and lesion-directed interactions, while integration with advanced formulations, such as microneedles, hydrogels, and microspheres, provides opportunities for localized delivery and controlled release. These strategies have been explored across various preclinical models, including AD, psoriasis, diabetic wounds, and photoaging, with reported antioxidant, immunomodulatory, antibacterial, and pro-angiogenic effects. Collectively, these studies suggest a potential evolution from simple antioxidant applications toward multifunctional therapeutic strategies characterized by immune-repair synergy.

Despite this potential, substantial barriers remain before CeO_2_-based nanotherapeutics can be considered ready for clinical translation. First, their long-term cutaneous fate and safety remain incompletely defined. In particular, systematic data on skin penetration and retention, physicochemical transformation, clearance, repeated-dose toxicity, and potential systemic exposure are still limited, especially under disease-relevant conditions with impaired barrier integrity. Second, reproducible and scalable manufacturing remains challenging because biological performance is highly sensitive to physicochemical characteristics such as particle size, morphology, surface chemistry, Ce^3+^/Ce^4+^ state, and oxygen-vacancy-related properties. Standardized material characterization, quality-control criteria, and batch-to-batch reproducibility will therefore be essential for meaningful comparison across studies and subsequent regulatory evaluation. Third, mechanistic and disease-specific evidence remains incomplete. Although antioxidant and downstream anti-inflammatory effects are relatively well documented in preclinical models, direct evidence linking defined CeO_2_ properties to specific immune pathways, cellular targets, and disease-dependent therapeutic responses remains limited. These uncertainties, together with the need to better understand microbiome interactions and formulation-dependent cutaneous exposure, currently preclude broad conclusions regarding clinical efficacy or long-term safety. Addressing these barriers will require coordinated progress in material standardization, biologically relevant safety assessment, mechanistic validation, and disease-specific preclinical evaluation.

**Figure 4:**
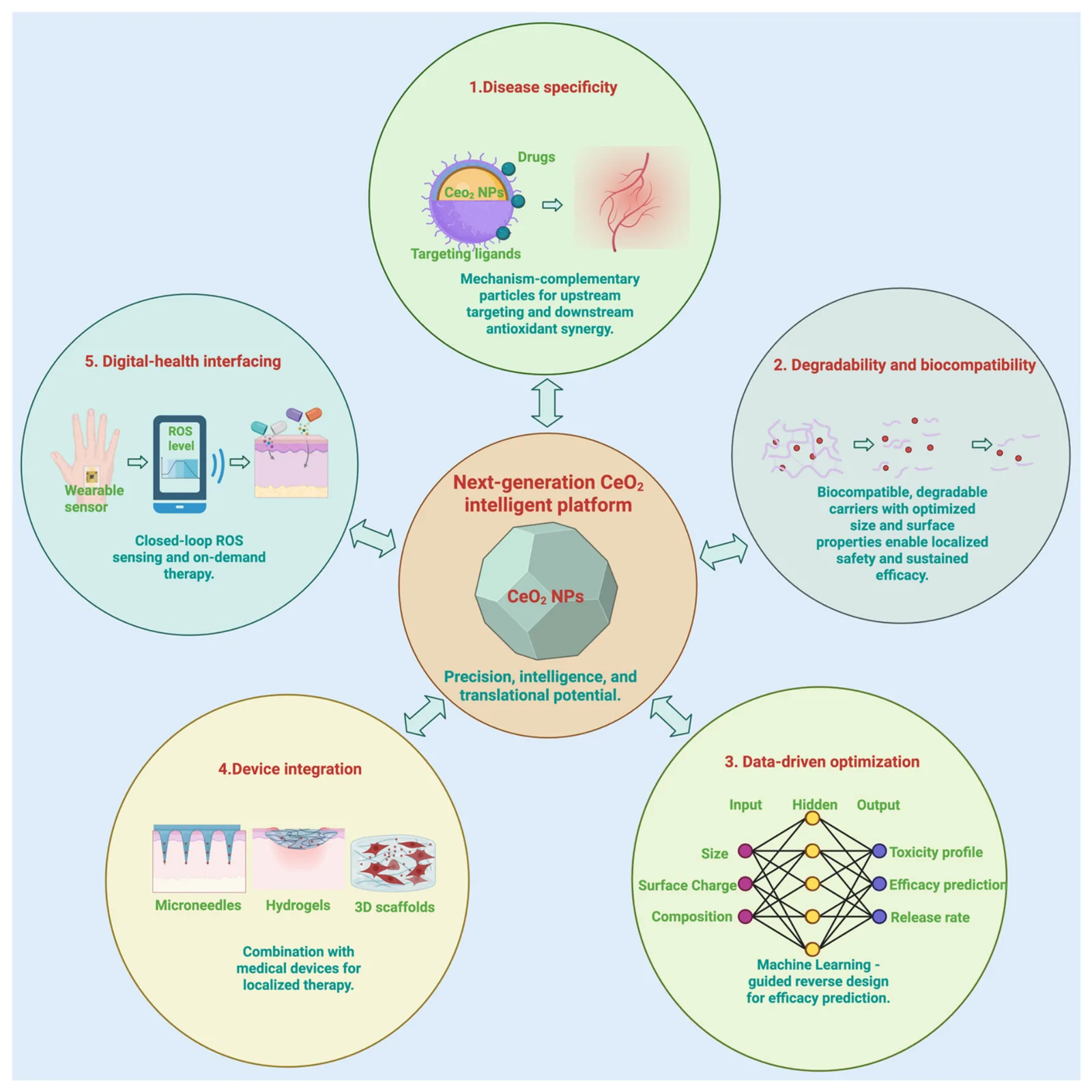
Schematic diagram of the intelligent design and future development directions of CeO_2_ NPs. This figure systematically illustrates five future-oriented design strategies for the use of CeO_2_ NPs in the treatment of inflammatory skin diseases: (1) disease specificity, which involves upstream targeting and downstream antioxidant synergy through targeting ligands and mechanism-complementary particles; (2) degradation and biocompatibility, in which biodegradable carriers with optimized size and surface properties are utilized to enable localized safety and sustained efficacy; (3) data-driven optimization, leveraging machine learning-guided reverse design to predict relationships among parameters such as particle size, surface charge, and composition with toxicity, efficacy, and release behavior; (4) device integration, in which CeO_2_ NPs are combined with medical devices such as microneedles, hydrogels, and three-dimensional scaffolds for localized therapy; and (5) digital-health interfacing, in which wearable sensors are employed to monitor ROS levels and establish a closed-loop sensing and on-demand drug delivery system, ultimately forming a next-generation intelligent platform based on CeO_2_. Created in BioRender. Liu, W. (2026) https://BioRender.com/lupab6d (accessed on 13 September 2026).

Looking ahead, the development of next-generation cerium oxide-based nanomedicines is poised to converge around a “5D” integration framework (Figure 4). To translate this conceptual framework into an actionable research roadmap, each dimension can be associated with specific technical priorities and validation milestones:

(1) Disease specificity. Material and combination design should be matched to the dominant pathological mechanisms of individual indications. For example, in immune-dominant inflammatory diseases such as AD and psoriasis, CeO_2_-mediated downstream redox regulation could be combined with therapies targeting upstream inflammatory pathways, such as JAK inhibitors or biologics, whereas infected or chronic wounds may require formulations that additionally incorporate antimicrobial and pro-reparative functions. Candidate strategies should subsequently be benchmarked against CeO_2_ alone and the corresponding conventional therapy in disease-relevant models, using predefined redox, inflammatory, barrier, microbial, and tissue-repair endpoints. Establishing such disease-specific performance profiles represents an important milestone for defining the indications in which CeO_2_-based combination strategies are most likely to provide added therapeutic value.
(2) Degradability and biocompatibility. The delivery vehicle and inorganic CeO_2_ core should be evaluated separately. Although biodegradable hydrogels, microspheres, and other carriers may facilitate local delivery and reduce carrier-associated persistence, their degradation does not ensure elimination of the CeO_2_ core. Development should therefore incorporate quantitative assessment of cutaneous retention, biotransformation, dissolution and cerium speciation, clearance kinetics, and repeated-dose toxicity. A key milestone will be the definition of an exposure window that maintains therapeutically useful local retention without undesirable persistent accumulation or unacceptable local or systemic toxicity.
(3) Data-driven optimization. A standardized structure–property–bioactivity database should be established before machine-learning-guided inverse design can be reliably implemented. Priority material descriptors should include particle size and size distribution, morphology and crystal characteristics, surface charge, Ce^3+^/Ce^4+^ ratio, oxygen-vacancy-related properties, surface modification, and dopant composition, because these parameters are major determinants of CeO_2_ catalytic and biological behavior. These descriptors should be linked to standardized outputs, including catalytic activity, cytotoxicity, inflammatory and immune responses, antimicrobial activity, cutaneous delivery and retention, and therapeutic efficacy. A practical workflow would progress from harmonized data generation to model training and internal validation, followed by validation against independent datasets. Model-predicted candidate formulations should then be prospectively synthesized and experimentally tested, with the resulting data incorporated into subsequent rounds of model refinement. Accordingly, practical milestones for data-driven development should include the establishment of standardized datasets, robust model training and internal validation, independent external validation, prospective experimental verification of model-predicted candidates, and iterative refinement based on newly generated data.
(4) Device integration. Platform selection should be guided by the intended delivery depth and therapeutic objective. Hydrogels are particularly suitable when prolonged surface contact and microenvironment-responsive release are required, whereas microneedles provide a rational option when CeO_2_ must be delivered across the stratum corneum into viable skin layers. Microspheres and vesicular carriers may instead be prioritized when sustained release, protection of nanozyme activity, or prolonged local retention is desired. Compatibility should be evaluated in terms of nanoparticle dispersion and stability, loading efficiency, delivery depth, dose reproducibility, release kinetics, and device performance. Reproducible delivery of a defined CeO_2_ dose to the intended cutaneous compartment without compromising either nanozyme activity or device performance should represent a key translational milestone.
(5) Digital-health interfacing. As the most forward-looking component of the 5D framework, digital-health integration should focus initially on establishing a reliable link between measurable skin signals and therapeutic decision-making. Wearable sensing platforms could be used to monitor disease-relevant parameters, such as local oxidative stress, and longitudinal changes in these signals could then be correlated with disease severity and treatment response. Once robust sensing-response relationships have been established, CeO_2_-based formulations could be integrated with externally controllable or stimuli-responsive delivery systems, allowing treatment intensity or release behavior to be adjusted according to the monitored skin microenvironment. The key technical milestones are therefore the identification of clinically informative biomarkers, validation of sensor accuracy and stability under realistic skin conditions, demonstration of reproducible coupling between sensor output and drug-release control, and ultimately evaluation of feedback-guided treatment in disease-relevant models. Such a stepwise strategy would provide a more realistic basis for progressing from passive wearable monitoring toward closed-loop, on-demand CeO_2_-based therapy.

Importantly, the 5D framework should be viewed as a research and translational roadmap rather than evidence of current clinical readiness. Although existing preclinical studies support the therapeutic potential of CeO_2_-based systems, their clinical translation will ultimately depend on reproducible manufacturing and standardized material characterization, rigorous assessment of cutaneous fate and repeated-dose safety, disease-specific mechanistic validation, and demonstration of therapeutic benefit in clinically relevant preclinical models and, subsequently, well-designed human studies. More advanced concepts, including machine-learning-guided material design and closed-loop delivery, should therefore be regarded as longer-term opportunities whose implementation depends on the establishment of robust experimental datasets and validated sensing and delivery technologies. Addressing these challenges will be critical for translating the distinctive redox activity, physicochemical tunability, and formulation versatility of CeO_2_ into safe, reproducible, and clinically meaningful therapeutic strategies for inflammatory skin diseases.

## Figures and Tables

**Table 1:** Comparison of preparation methods: principles, advantages, and limitations.

Methods	Principles	Advantages	Limitations	References
Complexation precipitation method	Citrate complexation followed by calcination	Simple operation, high specific surface area, and excellent UV-shielding properties	Complex precursor composition, requires precise control of conditions	[12]
Combustion synthesis method	Exothermic reaction between fuel and oxidizer	Rapid, straightforward, and cost-effective	Residual organic matter may remain, products are prone to agglomeration	[13]
Sol–gel method	Hydrolysis-condensation followed by calcination	High purity, uniform particle size, and tunable properties	Long processing time, high calcination temperature	[14]
Hydrothermal method	High-temperature, high-pressure hydrothermal reaction	Controllable morphology, high crystallinity, and suitable for doping	High equipment requirements, lengthy processing time, product properties are highly sensitive to precursor parameters	[15]
Reverse microemulsion method	Confined reaction within nanoscale aqueous cores	Uniform particle size, good monodispersity, strong metal-support interaction, excellent catalytic performance	High cost, complex synthesis process, low yield	[16]
Green precipitation method	Plant extracts for reduction and stabilization	Environmentally friendly, excellent biocompatibility, and high antibacterial activity	Poor reproducibility, limited morphological control	[17]

**Table 2:** Biomarkers and functions of various cell membranes.

Cell Membrane Types	Biomarker	Functions	References
Red blood cell membrane	CD47, CD59	Prolonging circulation and enhancing immunocompatibility by reducing immune recognition	[115,116]
Platelet membrane	CD47, CD55/59	Evading immune surveillance and preventing complement activation	[117]
Neutrophil membrane	LFA-1, integrin β1	Targeting inflammatory sites	[118]
Macrophage membrane	CCR2, integrins	Neutralizing inflammatory mediators, reducing reticuloendothelial system clearance, and prolonging systemic circulation	[113,119,120,121,122]
Dendritic cell membrane	PD-L2	Suppressing Th2 immune responses	[123]
Regulatory T-cell membrane	CD152, CD279	Mediating immunosuppression	[124]
CD4^+^ T-cell membrane	CD4	Blocking IL-23 signaling and suppressing Th17 differentiation by competitively binding IL-23	[114]
Mesenchymal stem cell membrane	CD29, IDO, TGF-β	Targeting inflamed tissues and suppressing T-cell activation	[125]

Abbreviations: CD, cluster of differentiation; LFA-1, lymphocyte function-associated antigen-1; CCR2, C-C chemokine receptor type 2; IDO, indoleamine 2,3-dioxygenase.

## Data Availability

No new data were created or analyzed in this study. Data sharing is not applicable to this article.

## Abbreviations

The following abbreviations are used in this manuscript:

CeO_2_ NPs	Cerium oxide nanoparticles
AD	Atopic dermatitis
SOD	Superoxide dismutase
CAT	Catalase
ROS	Reactive oxygen species
NF-κB	Nuclear factor kappa B
TNF-α	Tumor necrosis factor-alpha
IL	Interleukin
TGF-β	Transforming growth factor-beta
Th2	T helper 2
DNA	Deoxyribonucleic acid
VEGF	Vascular endothelial growth factor
HIF-1α	Hypoxia-inducible factor-1α
PDGF	Platelet-derived growth factor
MAPK	Mitogen-activated protein kinase
JAK	Janus kinase
JNK	c-Jun N-terminal kinase
PD-L1	Programmed death-ligand 1
CD	Cluster of differentiation
LFA-1	Lymphocyte function-associated antigen-1
CCR2	C-C chemokine receptor type 2
IDO	Indoleamine 2,3-dioxygenase
DBCO	Dibenzocyclooctyne
